# The Paradox of Feline Coronavirus Pathogenesis: A Review

**DOI:** 10.1155/2011/109849

**Published:** 2011-08-21

**Authors:** Luciana Wanderley Myrrha, Fernanda Miquelitto Figueira Silva, Ethel Fernandes de Oliveira Peternelli, Abelardo Silva Junior, Maurício Resende, Márcia Rogéria de Almeida

**Affiliations:** ^1^Laboratório de Infectologia Molecular Animal (LIMA), Universidade Federal de Viçosa, Avenida Peter Henry Rolfs, s/n Campus Universitário, 36570-000 Vicosa, MG, Brazil; ^2^Laboratório de Doença das Aves, Universidade Federal de Minas Gerais, Avenida Antônio Carlos, 6627—Pampulha, 31270-901, Belo Horizonte, MG, Brazil

## Abstract

Feline coronavirus (FCoV) is an enveloped single-stranded RNA virus, of the family *Coronaviridae* and the order *Nidovirales*. FCoV is an important pathogen of wild and domestic cats and can cause a mild or apparently symptomless enteric infection, especially in kittens. FCoV is also associated with a lethal, systemic disease known as feline infectious peritonitis (FIP). Although the precise cause of FIP pathogenesis remains unclear, some hypotheses have been suggested. In this review we present results from different FCoV studies and attempt to elucidate existing theories on the pathogenesis of FCoV infection.

## 1. Introduction

Feline coronavirus (FCoV) belongs to the family *Coronaviridae* and the order *Nidovirales* [1] and affects both wild and domestic cats [2]. FCoV contains a positive polarity RNA genome approximately 29 kb in length, consisting of 11 open reading frames (ORFs). Two major ORFs encode a replicase, four ORFs encode the structural proteins S (spike), E (envelope), M (membrane), and N (nucleocapsid), and five ORFs encode the nonstructural proteins 3a, 3b, 3c, 7a, and 7b [3].

FCoV can cause a mild or sometimes apparently symptomless enteric infection, especially in kittens, and is also associated with a lethal, systemic disease known as feline infectious peritonitis (FIP) [4, 5]. FIP is characterized by fibrinous, granulomatous serositis, with protein-rich effusions in the body cavities of affected cats (effusive or “wet” FIP), as well as granulomatous-necrotizing lesions, periphlebitis and granulomatous inflammatory lesions in several organs, especially, liver, kidney, spleen, leptomeninges, and eyes (noneffusive or “dry” FIP) [6].

Although the precise cause of FIP pathogenesis is still unknown, several hypotheses have been suggested. One assumption is that a mutant FCoV strain is able to infect monocytes and macrophages, leading to FIP [7–9]. This mutant virus strain has been named feline infectious peritonitis virus (FIPV), whereas the strain that causes enteric infection was named feline enteric coronavirus (FECV) [7, 10–12]. Because FIPV and FECV cannot be distinguished by their antigenicity, or even by genome sequence analysis, they are considered to be two, distinctly different pathotypes, which differ only in their pathogenicity [11, 12]. In fact, although this hypothesis is known as the internal mutation theory, no specific mutation has been identified in the 29 kb FCoV genome [13].

A second hypothesis for the development of FIP proposes that any FCoV strain can cause FIP disease. Instead, host factors, such as immune response variations [13–16], and viral factors, such as the formation of quasispecies [17], determine whether or not FIP develops.

Brown et al. [18] have proposed yet a third hypothesis for FIP pathogenesis. These authors suggest, after phylogenetic analyses, of the partial membrane gene and the partial nonstructural protein 7b gene from sequences from healthy cats and sick cats that, possibly, there are two different strains, virulent and avirulent, circulating in natural feline population and FIP development occurs when an animal is infected with the virulent strain.

Thus, to date, many experimental studies and different hypothesis have been reported in the literature attempting to explain FIP pathogenesis (Table 1). This review, therefore, presents these different research studies and attempts to elucidate existing theories on the pathogenesis of FCoV infection.

## 2. Internal Mutation Theory

The close similarity of FECV to FIPV, and the low incidence of FIP, despite the high proportion of FCoV seropositive cats, led to the hypothesis that FECV carriers are sources of FIPV, which is proposed to be generated by small mutations in FECV [5, 19]. After this hypothesis was first postulated in the literature, several studies have been conducted to test its validity.

Initially, this mutational theory was questioned, when the 3′ ends of the genomes (the corresponding ORF7a/7b) of an FIPV strain, which had been adapted to tissue culture (strain WSU 79-1146), and an FECV strain (strain WSU 79-1683) were genetically compared. FECV was shown to contain a 238-nucleotide deletion in ORF7b [20], in contrast to what might be expected if FIPV were indeed a mutant of FECV. According to Vennema et al. [20], it is possible that both FECV and avirulent FIPV strains are derived from FIP inducing strains but have been attenuated by the loss of virulence factors. The authors further propose that recombination of two attenuated viruses during a rare mixed infection event could result in the resurgence of a virulent FIPV strain. 

Evidence in support of the mutation theory came from comparative analysis of 1.2 kb genomic fragments obtained from nine additional FECV and FIPV isolates [21]. All of these isolates were found to have intact ORF7bs, indicating that the deletion observed in FECV 79-1683 is associated with adaptation to cell culture. These results are in agreement with previous studies that showed that four strains of FCoV (79-1683, TN406-HP, UCD2, and ts-DF2) with deletions in ORF7b were avirulent [22, 28]. Thus, it is likely that, for these viruses, ORF7b deletions contribute to the loss of virulence. Therefore, ORF7b seems to provide a distinct selective advantage during natural infection [21].

Because ORF7b deletions had been identified in previous studies, researchers began to assess the involvement of other regions of the genome in FCoV pathogenicity. Subsequent studies have shown that both deletions and nonsense mutations within ORF3c and, less often, specific mutations in OFR7b are present in FIPVs but not in FECVs [9]. Sequencing of attenuated derivatives of virulent FIPV strains shows good correlation between deletions in ORF7b and attenuation of virulence [21]. This latter observation indicates that the 7b protein encoded by ORF7b is important for virulence. Thus, the virulence-associated ORF7b may be somewhat suppressed in FECVs not adapted to tissue culture, and the presence of an intact ORF3c in these FECVs may be involved in this suppression. Moreover, two cats from the same household were found to contain distinct ORF3c deletions, demonstrating that these two FIPVs were independently derived from FECV and not transmitted horizontally [9].

Kennedy et al. [23] analyzed ORF7a and ORF7b in cheetah FCoVs over a period of 4 years. In most isolates, a deletion mutation was observed in ORF7a that results in an open reading frame change, possibly leading to expression of an aberrant ORF7a product, or loss of ORF7a expression. In addition, although specific mutations were also found in ORF7b, these mutations would not result in important changes within the resulting protein, suggesting that this protein may be essential for viral replication or infection [23].

Subsequent analysis of ORF7a and ORF7b revealed two distinct virus variants circulating in a population of 15 Persian cats: one with ORF7a intact and the other with two major deletions (nucleotides 20–120 and nucleotides 164–226) in ORF7a [24]. ORF7b was intact and similar among all isolates, but specific mutations resulted in changes in ORF7b protein amino acids. Some cats in this study were doubly infected with both viral variants. Because the cats were exposed to both variants, the authors concluded that there must be a causal relationship between the occurrence of these mutations and the increased incidence of FIP in this population [24]. According to these authors, in addition to the viral variant, disease variation must be related to host factors, such as the ability of the host to generate an effective immune response.

Alternatively, many authors have proposed that mutations in other genomic regions might be responsible for the variation in virulence observed in FCoV strains [8, 25]. Specifically, analysis of the 5′ hypervariable region of the S gene revealed consecutive accumulation of nucleotide substitutions nonsynonymous in this region, suggesting sequential emergence of viral variants, each time replacing the pre-existing virus population as a result of immune selection during chronic infection [25].

Because deletions in ORF7b were previously identified in laboratory strains with variable numbers of passages, these were considered to be associated with loss of virulence [20, 21, 29]. In contrast to these previous findings, sequence comparisons of FCoV ORF7bs isolated from some cats with FIP revealed small, in-frame deletions in the 3′ region of ORF7b, implying that the presence of this deletion is not correlated with FIP pathogenicity [30].

Studies have also shown that ORF3c presents great genetic variability in cats with FIP [2, 26]. The expression of functional ORF3c protein is crucial for FECV replication in the gut, but dispensable for systemic FIPV replication [26]. It was observed that ORF3c is intact in all strains of FECVs, but it is mutated in most FIPV strains. However, as some FIPVs seem to have intact ORF3c, it is likely that 3c mutations are not the only cause of FIP [2, 26].

To investigate the genetic differences that may result in the increased pathogenicity of FIPV with respect to FECV, Dye and Siddell [13] compared the complete sequences of viral RNA samples extracted from the liver and jejunum of a cat with classical FIP and observed 100% identity between them, calling into question the internal mutation theory. However, studies in support of this theory contend that, because deleterious ORF3c mutations tend to be found in the tissues of sick animals, while intact ORF3c is found mainly in feces [2], fecal samples of FIPV may be present as a result of extensive intestinal lesions, which could explain the presence of the FIPV in the jejunum in the experiments carried out by Dye and Siddell [13].

Sequence comparisons also show that FECVs and FIPVs taken from cats in the same geographical area are closely related, whereas there are significant genetic variations between FECVs and FIPVs from different geographic areas [2, 9]. According to these authors, the high genetic similarity between FIPV and FECV isolates from cats of the same geographic region strongly suggest a common ancestor. Furthermore, the occurrence of deleterious mutations in FIPVs, but not in FECVs, is believed to confirm the hypothesis that FIPVs emerged from FECVs.

The theory that FIPVs originated from mutations in FECVs was reinforced by studies of experimental infection. Cats experimentally infected with an FECV RM strain [7] remained asymptomatic during the first 2 months postinfection, but 8 to 10 weeks postinfection, 2 cats developed FIP. Viruses isolated from these two cats (FIPV-UCD9 and FIPV-UCD10) were found to have high sequence identity with each other and with FECV-RM and induced FIP when inoculated intraperitoneally in specific pathogen-free (SPF) cats [7]. This study showed that FIPVs can rapidly arise by mutations in FECVs and that these mutations frequently occur, although another study showed that cats persistently infected by FECV rarely develop FIP [31].

According to Kipar et al. [32], an increase in viral replication capacity may be a key feature in the development of FIP. Failure to control FCoV replication could lead to an increase in viral load, thus increasing the chances that a pathogenic mutation will be generated [25]. Furthermore, immunosuppression caused by infection with feline leukemia virus (FELV) or feline immunodeficiency virus (FIV) can reinforce the creation and selection of mutant FIPVs by increasing the rate of FECV replication in the gut and inhibiting the ability of the host to fight mutated viruses once they are formed. Thus, both viral and host factors determine the outcome of FCoV infection [4, 7].

## 3. Hypotheses Related Viral and Host Factors in the FCoV Pathogenesis

### 3.1. Quasispecies Theory

Viruses with RNA genomes have high mutation rates during development and larger viral genomes have higher mutation rates compared with smaller genomes. This fact, coupled with its rapid and continuous replication, allows for rapid production of viral genetic diversity, including mutations that facilitate adaptation to the host [33].

The high mutation rates in viruses with RNA genomes, which use RNA polymerase-dependent RNA, occur because of the absence of proofreading activity in this enzyme. In coronavirus, although its RNA polymerase has a domain with exoribonuclease 3′-5′ activity [34], a high number of errors still can occur in the genome, giving rise to a population of heterogeneous closely related sequences called quasispecies [17]. In the coronaviruses, quasispecies formation has been well documented for murine hepatitis virus (MHV) [35].

FCoV can also form quasispecies with significant genetic heterogeneity, as a result of accumulation of mutations during viral replication [17, 27, 36]. These viral subpopulations were analyzed by single-strand conformational polymorphism (SSCP), for polymerase chain reactions (PCR) products of genes N and 7b [17, 36]. These studies demonstrate that the composition of FCoV quasispecies in a single cat can differ in different organs [17, 36], and that the heterogeneity of the FCoV genome is related to the severity and clinical form of FIP and the lesions observed in the organs of affected animals [17]. New viral strains present in these subpopulations can alter cell tropism and pathogenicity and may have a significant impact on generating host disease [13]. However, it still remains unclear if the association between genetic diversity and pathogenesis can be attributed to quasispecies dynamics [33]. Moreover, the mere observation of high levels of genetic variation in RNA viruses is not proof of the existence of quasispecies. To demonstrate that RNA viruses form quasispecies, it is necessary to prove that natural selection acts on the viral population as a unit [37].

### 3.2. Immune Response Related to Viral-Host Interaction

An important feature of FCoV pathogenesis is the intrinsic resistance of macrophages to FCoV infection [38]. It has been postulated that the emergence of highly virulent FIPV biotypes from FECV is accompanied by a dramatic change in cell tropism that allows FIPV to infect monocytes and then be disseminated systemically [8, 10, 39].

Macrophages infected with FIPV play a key role in the immunopathological damage observed in FIP. These cells are the most predominant inflammatory cells in FIP lesions [40] and viral antigens can be detected in macrophages isolated from pyogranulomatous lesions [41] and in monocytes (progenitors of tissue macrophages) isolated from effusions [42]. Moreover, the decrease of CD4 T cells and, especially, CD8 T cells, as a result of apoptosis is probably related to tumor necrosis factor alpha (TNF-alpha) released by macrophages infected with FCoV [11].

Probably, FIPV replicates in monocytes and macrophages because these cells do not express viral antigens on their surface, creating a form of escape through the humoral immune system [43]. However, FIPV-like cell tropism by macrophages is also observed for FECV, because this biotype is also capable of infecting monocytes and macrophages [14, 44]. Furthermore, cats that do not present with any clinical or pathological evidence of FIP can maintain viremia in their monocytes for a period of 3 to 12 months [16, 36, 44], indicating that the development of FIP is not dependent on the systemic spread of FCoV [16] and that the susceptibility of monocytes/macrophages to FCoV infection is only one of the pathogenic events responsible for FIP. Kipar et al. [32] showed that, independent of the development of FIP, infection by FCoV induces proliferation and activation of monocytes/macrophages.

Host genetic factors are also important for the susceptibility of monocytes to FCoV infection. Studies in vitro showed that monocytes from different cats do not have the same susceptibility to FCoV infection (by the same strain of FCoV) suggesting that cellular factors, influenced by genetic background and/or differentiation/activation status, are very important in determining the occurrence of FIP [14, 45]. This resistance to FCoV infection seems to occur also in natural infections. A study in vivo showed that a small percentage of cats in FCoV endemic households had no shedding and remained seronegative or had a low antibody titer over a time period of 5 years [46].

An ineffective immune response against FCoV infection seems to be an important factor in FIP pathogenesis [36]. It has been hypothesized that animals with a weak cell-mediated immunity (CMI) in combination with a strong humoral immune response are likely to develop FIP and cats with a strong CMI may not develop the disease [47].

There are indications that a strong humoral immune system plays an adverse role in the development of FIP [48–50]. The antibody titer, in FIPV infection, is not effective for elimination of the virus, and, inversely, they enhance FIP development in vitro [48] and in vivo, in cats previously immunized against FCoV [50, 51]. The phenomenon antibody-dependent enhancement (ADE) could explain this accelerated development of FIP in the presence of antibodies. In ADE, antibodies might help the spread of the virus in an infected cat by facilitating the virus uptake through the formation of virus-antibody complexes that are taken up by uninfected monocytes/macrophages via the Fc receptor [49]. A recent study in vitro showed that viral plasma membrane-bound proteins of FCoV (S and M) were internalized by monocytes upon antibody addition [52].

Differently, it has been proposed that ADE does not occur in cats with strong CMI, even if they possess anti-FCoV antibodies, escaping from FIP development [50]. 

Significant differences found in the composition and functional state of lymphatic tissues from FcoV-infected cats, with and without clinical signs of FIP, have been proposed to play an important role in FIP pathogenesis [32, 45, 53, 54].

Cats infected with FCoV, but without clinical signs of FIP, generally exhibit B and T cells hyperplasia with high lymphocyte proliferation [53–55] and exhibit higher expression levels of feline interleukin- (IL-) 10 in the spleen, as shown by quantitative real-time PCR (qRT-PCR). In addition, these cats have decreased IL-6 levels [32]. In contrast, Takano et al. [56] showed that peripheral blood mononuclear cells (PBMCs) from FIP cats displayed higher IL-6 expression compared to the same cells from SPF cats and suggest that IL-6 is involved in the development of immune-complex-mediated vasculitis and, therefore, in FIP pathogenesis. This probably occurs due to the action of IL-6 to recruit and activate T cells and macrophages, to expand cytotoxic T lymphocytes, to modulate the differentiation of plasma cells and promote increase of vascular permeability [57]. In the central nervous system IL-6 contributes to immune-mediated destruction observed in this tissue from cats with neurological clinical sings of FIP [57].

IL-6 is negatively regulated by IL-10 [58]. IL-10 stimulates Natural killer cells positively regulates the expression of Fc*γ* I receptors and antibody-dependent cellular cytotoxicity. This cytotoxicity probably contributes to viral elimination [59] and to the low viral loads observed in asymptomatic, FCoV carrier cats [15]. However, another study reported that high viral load is not related to the development of FIP [16]. Furthermore, IL-10 negatively regulates the expression of *β*2-integrins on monocytes, reducing their ability to adhere to endothelial cells and causing vasculitis [6]. However, the role of IL-10 in protection against FIP requires further study, because Dean and coauthors [60] observed high IL-10 expression levels in tissues of cats infected with a highly pathogenic FIPV strain (FIPV UCD8).

Lymphoid depletion has been reported in lymphatic tissues [54, 60] and in blood [55] of FcoV-infected animals, with clinical signs of FIP. This lymphoid depletion is likely due to apoptosis, probably mediated by TNF-alpha release, infected macrophages, and significantly decreased IL-12 p40 expression [32, 60]. This decrease in IL-12 p40 coupled with the presence of large numbers of activated macrophages in the lymphatic tissue and granulomatous infiltrates are signs of immune response failure [32]. IL-12 and Interferon- (IFN-) gamma coordinate the link between pathogen recognition by innate immune cells and the induction of specific immunity, by mediating a positive feedback to amplify the Th1 response (cell-mediated). IFN-gamma is an important cytokine for the Th1 immune response by inducing effector mechanisms such as innate cell-mediated immunity, specific cytotoxic immunity, and macrophage activation [61]. 

Cytokine mRNA measurements from PBMC from cats previously immunized with an avirulent FIP strain (FIPV-UCD1) and then challenge-exposed to a highly virulent cat passaged strain (FIPV-UCD8) with classical effusive FIP and noneffusive FIP showed that disease, regardless of form, is associated with a strong TNF-alpha mRNA response in PBMC and a failure to induce IFN-gamma mRNA. In contrast, asymptomatic cats of the same study failed to upregulate TNF-alpha mRNA and were observed in one asymptomatic cat strong IFN-gamma mRNA responses [62]. 

TNF-alpha mRNA response tends to favor Th2 immunity (humoral), while the IFN-gamma mRNA response favors Th1 immunity [62]. In FCoV-endemic cattery without a case of FIP the percentage of clinically healthy FCoV-positive cats expressing IFN-gamma is significantly high, suggesting that this cytokine, together with IL-1*β*, might protect infected cats from the disease [63].

Differently, another study showed high levels of IFN-gamma mRNA in tissues with inflammatory lesions of FIP indicating that the infection is not controlled only by the inflammatory response [40]. According to these authors, probably, cytokine profiles that run on samples from tissues with relevant inflammatory lesions could reflect the local cytokine response more adequately than in PBMC. A study with cats naturally infected with FCoV with clinical FIP and FCoV-infected clinically normal animals showed that cats with FIP do not have increased serum IFN-gamma concentrations [64] suggesting low IFN-gamma expression by PBMC like that mentioned by Berg et al. [40] or depleted numbers of lymphocytes in the blood or lymph nodes [54]. 

Moreover, cats naturally infected with FCoV, with clinical effusive FIP, had IFN-gamma concentrations in the effusions 40-fold higher than the serum concentrations of this cytokine in the same animals [64]. This suggests that the IFN-gamma present in the effusions is produced by cells within FIP lesions, as reported by Berg et al. [40].

A recent study with clinically normal cats naturally infected with FCoV and cats with effusive FIP showed high serum IFN-gamma concentrations in cats clinically normal and high IFN-gamma concentration of the FIP effusions. This suggests that although cats resistant to FCoV infection have strong CMI as measured by serum IFN gamma production, CMI is also likely to be involved in the pathogenesis of FIP, albeit at a tissue level, as evidenced by the high IFN-gamma concentration of the FIP effusions [64].

Another important factor involved in the pathogenesis of FIP is systemic inflammatory reaction. Acute phase proteins (APP) are plasma proteins produced by hepatocytes during systemic inflammation [65]. The major feline APP is *α*1-acid glycoprotein (AGP) [66]. In humans AGP is overexpressed during systemic inflammatory responses and its function appears to be related to an immunomodulatory activity [65].

In cats with FIP there has been observed increase of AGP concentration [67–69] and this AGP is hyposialylated (decrease in the degree of sialylation, a posttranslational modification) [68]. The decrease in the degree of sialylation is important for development of FIP. In catteries with endemic FCoV all cats respond to increased viral burden by increasing the production of AGP but only cats with hyposialylated AGP have persistently increased AGP levels and develop FIP [67]. Paltrinieri et al. [70] investigated the sialylation pattern of serum AGP in nonsymptomatic cats infected by FCoV and its relationship with the amount of FCoVs shed in faeces and observed that hypersialylation of AGP may be one of the factors that could explain why FCoV-infected cats do not develop FIP in spite of the presence of large amount of viral RNA shed in the environment. Possibly, the hypersialylation provides protection from the development of FIP [70].

### 3.3. Distinctive Circulating Virulent and Avirulent Strains

Recently, Brown et al. [18] formulated a new hypothesis explaining FIP pathogenesis, which suggests the existence of two different strains, virulent and avirulent, circulating among feline populations. These authors performed phylogenetic analysis of partial sequences from ORFs M, S, 3c, and 7b, isolated from FCoV carrier cats, with and without clinical signs of FIP. From this analysis, the authors inferred that FIPV and FECV form monophyletic groups, with high bootstrap values that clearly differentiate them genetically, supporting the presence of two circulating strains. However, more studies are needed using sequences from different geographic regions. In addition, further investigation is required into the correlation of these strains with host related factors, such as immune response, to confirm this hypothesis.

## 4. Conclusion

Despite numerous efforts by the scientific community to understand FIP pathogenesis, this disease still remains an enigma. As demonstrated in this review, the causes underlying FIP pathogenesis are probably multifactorial, with both viral and host factors as well as viral genetic determinants playing important roles in FIP pathogenesis. The studies necessary to show this interaction will likely be complex. Because FCoV is an RNA virus, it is able to easily mutate, and thus there are many FCoV viral subpopulations with variations in different regions of its genome. In addition, individual cats may respond differently to FCoV infection, suggesting that the immune response to FIP is complex. Thus, by definition, both of the FIP pathogenesis theories presented in this review are too simplistic, and further studies are essential to elucidate FIP pathogenesis, and to obtain information that will assist in the development of more accurate diagnostic methods and effective vaccines.

## Figures and Tables

**Table 1:** Hypotheses regarding the FIP pathogenesis.

Theory	References
Internal mutation theory	[2, 5, 7, 9, 19–26]
Quasispecies theory	[13, 17, 27]
Immune response related to viral-host interaction	[6, 11, 14, 16, 32, 36, 40–46, 48–60, 62–64, 67–70]
Distinctive circulating virulent andavirulent strains	[18]

## References

[1] De Vries AAF, Horzinek MC, Rottier PJM, De Groot RJ (1997). The genome organization of the nidovirales: similarities and differences between arteri-, toro-, and coronaviruses. *Seminars in Virology*.

[2] Pedersen NC (2009). A review of feline infectious peritonitis virus infection: 1963–2008. *Journal of Feline Medicine and Surgery*.

[3] Dye C, Siddell SG (2005). Genomic RNA sequence of Feline coronavirus strain FIPV WSU-79/1146. *Journal of General Virology*.

[4] Pedersen NC (1987). Virologic and immunologic aspects of feline infectious peritonitis virus infection. *Advances in Experimental Medicine and Biology*.

[5] Pedersen NC (1989). Animal virus infections that defy vaccination: equine infectious anemia, caprine arthritis-encephalitis, maedi-visna, and feline infectious peritonitis. *Advances in Veterinary Science and Comparative Medicine*.

[6] Kipar A, May H, Menger S, Weber M, Leukert W, Reinacher M (2005). Morphologic features and development of granulomatous vasculitis in feline infectious peritonitis. *Veterinary Pathology*.

[7] Poland AM, Vennema H, Foley JE, Pedersen NC (1996). Two related strains of feline infectious peritonitis virus isolated from immunocompromised cats infected with a feline enteric coronavirus. *Journal of Clinical Microbiology*.

[8] Rottier PJM, Nakamura K, Schellen P, Volders H, Haijema BJ (2005). Acquisition of macrophage tropism during the pathogenesis of feline infectious peritonitis is determined by mutations in the feline coronavirus spike protein. *Journal of Virology*.

[9] Vennema H, Poland A, Foley J, Pedersen NC (1998). Feline infectious peritonitis viruses arise by mutation from endemic feline enteric coronaviruses. *Virology*.

[10] Pedersen NC, Boyle JF, Floyd K, Fudge A, Barker J (1981). An enteric coronavirus infection of cats and its relationship to feline infectious peritonitis. *American Journal of Veterinary Research*.

[11] Takano T, Hohdatsu T, Hashida Y, Kaneko Y, Tanabe M, Koyama H (2007). A “possible” involvement of TNF-alpha in apoptosis induction in peripheral blood lymphocytes of cats with feline infectious peritonitis. *Veterinary Microbiology*.

[12] Vennema H (1999). Genetic drift and genetic shift during feline coronavirus evolution. *Veterinary Microbiology*.

[13] Dye C, Siddell SG (2007). Genomic RNA sequence of feline coronavirus strain FCoV C1Je. *Journal of Feline Medicine and Surgery*.

[14] Dewerchin HL, Cornelissen E, Nauwynck HJ (2005). Replication of feline coronaviruses in peripheral blood monocytes. *Archives of Virology*.

[15] Kipar A, Baptiste K, Barth A, Reinacher M (2006). Natural FCoV infection: cats with FIP exhibit significantly higher viral loads than healthy infected cats. *Journal of Feline Medicine and Surgery*.

[16] Meli M, Kipar A, Müller C (2004). High viral loads despite absence of clinical and pathological findings in cats experimentally infected with feline coronavirus (FCoV) type I and in naturally FCoV-infected cats. *Journal of Feline Medicine and Surgery*.

[17] Battilani M, Coradin T, Scagliarini A (2003). Quasispecies composition and phylogenetic analysis of feline coronaviruses (FCoVs) in naturally infected cats. *FEMS Immunology and Medical Microbiology*.

[18] Brown MA, Troyer JL, Pecon-Slattery J, Roelke ME, O’Brien SJ (2009). Genetics and pathogenesis of feline infectious peritonitis virus. *Emerging Infectious Diseases*.

[19] Pedersen NC, Evermann JF, McKeirnan AJ, Ott RL (1984). Pathogenicity studies of feline coronavirus isolates 79-1146 and 79-1683. *American Journal of Veterinary Research*.

[20] Vennema H, Rossen JWA, Wesseling J, Horzinek MC, Rottier PJM (1992). Genomic organization and expression of the 3’ end of the canine and feline enteric coronaviruses. *Virology*.

[21] Herrewegh AAPM, Vennema H, Horzinek MC, Rottier PJM, De Groot RJ (1995). The molecular genetics of feline coronaviruses: comparative sequence analysis of the ORF7a/7b transcription unit of different biotypes. *Virology*.

[22] Pedersen NC, Black JW (1983). Attempted immunization of cats against feline infectious peritonitis, using avirulent live virus or sublethal amounts of virulent virus. *American Journal of Veterinary Research*.

[23] Kennedy MA, Moore E, Wilkes RP, Citino SB, Kania SA (2006). Analysis of genetic mutations in the 7a7b open reading frame of coronavirus of cheetahs (Acinonyx jubatus). *American Journal of Veterinary Research*.

[24] Kennedy M, Boedeker N, Gibbs P, Kania S (2001). Deletions in the 7a ORF of feline coronavirus associated with an epidemic of feline infectious peritonitis. *Veterinary Microbiology*.

[25] Herrewegh AAPM, Mähler M, Hedrich HJ (1997). Persistence and evolution of feline coronavirus in a closed cat-breeding colony. *Virology*.

[26] Chang HW, de Groot RJ, Egberink HF, Rottier PJM (2010). Feline infectious peritonitis: insights into feline coronavirus pathobiogenesis and epidemiology based on genetic analysis of the viral 3c gene. *Journal of General Virology*.

[27] Gunn-Moore DA, Gunn-Moore FJ, Gruffydd-Jones TJ, Harbour DA (1999). Detection of FCoV quasispecies using denaturing gradient gel electrophoresis. *Veterinary Microbiology*.

[28] Christianson KK, Ingersoll JD, Landon RM, Pfeiffer NE, Gerber JD (1989). Characterization of a temperature sensitive feline infectious peritonitis coronavirus. *Archives of Virology*.

[29] Haijema BJ, Volders H, Rottier PJM (2003). Switching species tropism: an effective way to manipulate the feline coronavirus genome. *Journal of Virology*.

[30] Lin CN, Su BL, Huang HP, Lee JJ, Hsieh MW, Chueh LL (2009). Field strain feline coronaviruses with small deletions in ORF7b associated with both enteric infection and feline infectious peritonitis. *Journal of Feline Medicine and Surgery*.

[31] Pedersen NC, Allen CE, Lyons LA (2008). Pathogenesis of feline enteric coronavirus infection. *Journal of Feline Medicine and Surgery*.

[32] Kipar A, Meli ML, Failing K (2006). Natural feline coronavirus infection: differences in cytokine patterns in association with the outcome of infection. *Veterinary Immunology and Immunopathology*.

[33] Holmes EC (2009). The evolutionary genetics of emerging viruses. *Annual Review of Ecology, Evolution, and Systematics*.

[34] Minskaia E, Hertzig T, Gorbalenya AE (2006). Discovery of an RNA virus 3′ → 5′ exoribonuclease that is critically involved in coronavirus RNA synthesis. *Proceedings of the National Academy of Sciences of the United States of America*.

[35] Makino S, Keck JG, Stohlman SA, Lai MMC (1986). High-frequency RNA recombination of murine coronaviruses. *Journal of Virology*.

[36] Kiss I, Kecskeméti S, Tanyi J, Klingeborn B, Belák S (2000). Preliminary studies on feline coronavirus distribution in naturally and experimentally infected cats. *Research in Veterinary Science*.

[37] Moya A, Holmes EC, González-Candelas F (2004). The population genetics and evolutionary epidemiology of RNA viruses. *Nature Reviews Microbiology*.

[38] Morahan PS, Connor JR, Leary KR (1985). Viruses and the versatile macrophage. *British Medical Bulletin*.

[39] Stoddart CA, Scott FW (1989). Intrinsic resistance of feline peritoneal macrophages to coronavirus infection correlates with in vivo virulence. *Journal of virology*.

[40] Berg AL, Ekman K, Belák S, Berg M (2005). Cellular composition and interferon-*γ* expression of the local inflammatory response in feline infectious peritonitis (FIP). *Veterinary Microbiology*.

[41] Kipar A, Bellmann S, Kremendahl J, Köhler K, Reinacher M (1998). Cellular composition, coronavirus antigen expression and production of specific antibodies in lesions in feline infectious peritonitis. *Veterinary Immunology and Immunopathology*.

[42] Paltrinieri S, Parodi MC, Cammarata G (1999). In vivo diagnosis of feline infectious peritonitis by comparison of protein content, cytology, and direct immunofluorescence test on peritoneal and pleural effusions. *Journal of Veterinary Diagnostic Investigation*.

[43] Cornelissen E, Dewerchin HL, Van Hamme E, Nauwynck HJ (2007). Absence of surface expression of feline infectious peritonitis virus (FIPV) antigens on infected cells isolated from cats with FIP. *Veterinary Microbiology*.

[44] Gunn-Moore DA, Gruffydd-Jones TJ, Harbour DA (1998). Detection of feline coronaviruses by culture and reverse transcriptase-polymerase chain reaction of blood samples from healthy cats and cats with clinical feline infectious peritonitis. *Veterinary Microbiology*.

[45] Tekes G, Hofmann-Lehmann R, Bank-Wolf B, Maier R, Thiel HJ, Thiel V (2010). Chimeric feline coronaviruses that encode type II spike protein on type I genetic background display accelerated viral growth and altered receptor usage. *Journal of Virology*.

[46] Addie DD, Jarrett O (2001). Use of a reverse-transcriptase polymerase chain reaction for monitoring the shedding of feline coronavirus by healthy cats. *Veterinary Record*.

[47] Pedersen NC (1995). An overview of feline enteric coronavirus and infectious peritonitis virus infections. *Feline Practice*.

[48] Petersen NC, Boyle JF (1980). Immunologic phenomena in the effusive form of feline infectious peritonitis. *American Journal of Veterinary Research*.

[49] Hohdatsu T, Nakamura M, Ishizuka Y, Yamada H, Koyama H (1991). A study on the mechanism of antibody-dependent enhancement of feline infectious peritonitis virus infection in feline macrophages by monoclonal antibodies. *Archives of Virology*.

[50] Takano T, Kawakami C, Yamada S, Satoh R, Hohdatsu T (2008). Antibody-dependent enhancement occurs upon re-infection with the identical serotype virus in feline infectious peritonitis virus infection. *Journal of Veterinary Medical Science*.

[51] Vennema H, De Groot RJ, Harbour DA (1990). Early death after feline infectious peritonitis virus challenge due to recombinant vaccinia virus immunization. *Journal of Virology*.

[52] Dewerchin HL, Cornelissen E, Nauwynck HJ (2006). Feline infectious peritonitis virus-infected monocytes internalize viral membrane-bound proteins upon antibody addition. *Journal of General Virology*.

[53] Kipar A, Bellmann S, Gunn-Moore DA (1999). Histopathological alterations of lymphatic tissues in cats without feline infectious peritonitis after long-term exposure to FIP virus. *Veterinary Microbiology*.

[54] Kipar A, Köhler K, Leukert W, Reinacher M (2001). A comparison of lymphatic tissues from cats with spontaneous feline infectious peritonitis (FIP), cats with FIP virus infection but no FIP, and cats with no infection. *Journal of Comparative Pathology*.

[55] Paltrinieri S, Ponti W, Comazzi S, Giordano A, Poli G (2003). Shifts in circulating lymphocyte subsets in cats with feline infectious peritonitis (FIP): pathogenic role and diagnostic relevance. *Veterinary Immunology and Immunopathology*.

[56] Takano T, Azuma N, Hashida Y, Satoh R, Hohdatsu T (2009). B-cell activation in cats with feline infectious peritonitis (FIP) by FIP-virus-induced B-cell differentiation/survival factors. *Archives of Virology*.

[57] Foley JE, Rand C, Leutenegger C (2003). Inflammation and changes in cytokine levels in neurological feline infectious peritonitis. *Journal of Feline Medicine and Surgery*.

[58] Hannen M, Banning U, Bönig H (1999). Cytokine-mediated regulation of granulocyte colony-stimulating factor production. *Scandinavian Journal of Immunology*.

[59] Moore MA (2001). Interleukin-10 and the interleukin-10 receptor. *Annual Review of Immunology*.

[60] Dean GA, Olivry T, Stanton C, Pedersen NC (2003). In vivo cytokine response to experimental feline infectious peritonitis virus infection. *Veterinary Microbiology*.

[61] Boehm U, Klamp T, Groot M, Howard JC (1997). Cellular responses to interferon-*γ*. *Annual Review of Immunology*.

[62] Kiss I, Poland AM, Pedersen NC (2004). Disease outcome and cytokine responses in cats immunized with an avirulent feline infectious peritonitis virus (FIPV)-UCD1 and challenge-exposed with virulent FIPV-UCD8. *Journal of Feline Medicine and Surgery*.

[63] Gelain ME, Meli M, Paltrinieri S (2006). Whole blood cytokine profiles in cats infected by feline coronavirus and healthy non-FCoV infected specific pathogen-free cats. *Journal of Feline Medicine and Surgery*.

[64] Giordano A, Paltrinieri S (2009). Interferon-*γ* in the serum and effusions of cats with feline coronavirus infection. *Veterinary Journal*.

[65] Van Deventer SJH, Buller HR, Ten Cate JW, Aarden LA, Hack CE, Sturk A (1990). Experimental endotoxemia in humans: analysis of cytokine release and coagulation, fibrinolytic, and complement pathways. *Blood*.

[66] Cerón JJ, Eckersall PD, Martínez-Subiela S (2005). Acute phase proteins in dogs and cats: current knowledge and future perspectives. *Veterinary Clinical Pathology*.

[67] Paltrinieri S, Metzger C, Battilani M, Pocacqua V, Gelain ME, Giordano A (2007). Serum *α*1-acid glycoprotein (AGP) concentration in non-symptomatic cats with feline coronavirus (FCoV) infection. *Journal of Feline Medicine and Surgery*.

[68] Ceciliani F, Grossi C, Giordano A, Pocacqua V, Paltrinieri S (2004). Decreased sialylation of the acute phase protein *α*1-acid glycoprotein in feline infectious peritonitis (FIP). *Veterinary Immunology and Immunopathology*.

[69] Duthie S, Eckersall PD, Addie DD, Lawrence CE, Jarrett O (1997). Value of *α*1-acid glycoprotein in the diagnosis of feline infectious peritonitis. *Veterinary Record*.

[70] Paltrinieri S, Gelain ME, Ceciliani F, Ribera AM, Battilani M (2008). Association between faecal shedding of feline coronavirus and serum *α*1-acid glycoprotein sialylation. *Journal of Feline Medicine and Surgery*.

